# The improvement of eggs quality of Mojosari duck (*Anas javanica*) with soybean husk fermentation using cellulolytic bacteria of *Spodoptera litura*

**DOI:** 10.14202/vetworld.2018.720-725

**Published:** 2018-05-29

**Authors:** Sri Hidanah, Dady Soegianto Nazar, Erma Safitri

**Affiliations:** 1 Department of Animal Husbandry, Faculty of Veterinary Medicine, Universitas Airlangga, Surabaya, Indonesia; 2 Department of Veterinary Reproduction, Faculty of Veterinary Medicine, Universitas Airlangga, Surabaya, Indonesia; 3 Stem Cells Research Division of Institute Tropical Disease (ITD), Universitas Airlangga, Surabaya, Indonesia

**Keywords:** cellulolytic bacteria, eggs quality of duck, soybean husk fermentation, *Spodoptera litura*

## Abstract

**Aim:**

This study was aimed to improve the quality of the eggs of Mojosari duck (*Anas javanica*) through complete feeding containing soybean husk was fermented using cellulolytic bacteria of *Spodoptera litura*.

**Materials and Methods:**

This study consisted of three stages: The first stages, isolation and identification of cellulolytic bacteria from *S. litura*; the second stage, the fermentation of soybean husk through the application of bacterial cellulolytic isolate from the first stage; and the third stage, the application of the best complete feed formulation from the second stage to Mojosari duck.

**Results:**

There are four dominant bacteria: *Bacillus* sp., *Cellulomonas* sp., *Pseudomonas* sp., and *Cytophaga* sp. Furthermore, the best reduction of the crude fiber of soybean husks is the use of *Cellulomonas* sp. bacteria. The final of the study, the quality of the eggs of *Anas javanica*, was improved, as indicated by cholesterol decrease from the yolk without the decrease of egg weight and eggshell thickness, although the decrease in egg yolk color was inevitable.

**Conclusion:**

Soy husk fermentation using cellulolytic bacteria of *S. litura* was added to complete feeding can be performed to improve the quality of the eggs of Mojosari duck.

## Introduction

Soybean is an agricultural product that has been utilized to meet the needs of industry and food, such as Tempe, tofu, soy sauce, and soy milk. In general, the use and utilization of soybean are limited to seeds only, while the waste, such as soybean husk, is still discarded and has not been widely utilized. Analysis of dry matter (DM)=91.11, crude protein (CP)=5.04, ether extract=1.65, nitrogen-free extract, calcium=21, phosphorus=0.06, and gross energy=(kcal/g. DM) 3.98 according to the methods described in AOAC. The analysis of neutral detergent fiber=60.15 and acid detergent fiber=42.08 was carried out according to detergent method [1]. In other research, the chemical composition of soybean husk comprises 47.01% crude fiber, 14.45% CP, 3.04% crude fat, 3.15% ash, and 3.060, 48 kcal kg of energy metabolism. Soybean husk contains 42-49% dry weight of cellulose, 29-34% hemicellulose, and 1-3% lignin and has anti-nutritional antitrypsin substances [2].

On the other hand, *Spodoptera litura* is a pest of soybean crop that has a very high ability in damaging the plant. The leaves and pods attacked by *S. litura* become holes even then torn [3]. Based on its ability to damage the leaves and pods, allegedly the digestive tract of *S. litura* contains cellulolytic bacteria capable of digesting crude fibers well [4].

In general, cellulolytic bacteria have three cellulose enzymes called endoglucanase or carboxymethylcellulose (CMC-ase), exoglucanase or cellobiohydrolase, and beta-glucosidase. The enzymes can degrade cellulose into glucose [5]. CMC-ase breaks the hydrogen bonds present in the cellulose crystalline structure, forming single cellulose chains. Exoglucanase cuts off the ends of single chains cellulose, producing disaccharides and tetrasaccharides, cellobiose, beta-glucosidase hydrolyzes disaccharides, and tetrasaccharides into glucose [6]. Therefore, the utilization of cellulolytic microbes in the fermentation process of the feed material from the waste can allegedly improve the quality of complete feed formulation with the indication of the decrease of crude fiber and the increase of CP.

Based on another study, the soybean husk waste fermented with *Aspergillus niger* and Lactobacillus was only able to decrease crude fiber from 44% to 40%. The decrease in crude fiber content is still relatively small. In addition to the decrease in crude fiber, the fermentation process is also expected to increase CP from processed waste material [7]. Therefore, we need an alternative bacterium that has the higher capability in breaking down crude fiber along with an increase in CP content of the soybean husk.

This study aims to determine the potential of cellulolytic bacteria was contained in *S. litura* as a source of probiotics that can reduce the soybean crude fiber derived from the Tempe (Tempe is a traditional soy product originating from Indonesia. It is made by a natural culturing and controlled fermentation process that binds soybeans into a cake form) industry through the fermentation process, but followed by increased CP. If this is realized, then the quality of complete feed formulation on feed given to *Anas javanica* will be improved. Furthermore, improving the quality of complete feed formulation on feed was given to *Anas javanica* is expected to affect the quality of the eggs produced, such as low cholesterol levels with maintaining eggs’ weight, yolk color, and thickness of the shell.

## Materials and Methods

### Ethical approval

The present study was approved by ethical committee vide Ethical Clearance KE (Komisi Etik Penelitian), Animal Care and Use Committee (ACUC). Veterinary Medicine Faculty, Universitas Airlangga, Surabaya, Indonesia.

### Stage of study

This study consisted of three stages.

#### First stage

The first stage, isolation and identification of cellulolytic bacteria from *S. litura* digestive tract [4,8]; in total, 4 bacteria, i.e., *Bacillus* sp., *Cellulomonas* sp., *Pseudomonas* sp., and *Cytophaga* sp.were characterized based on their colony color, morphological, biochemical, and molecular characteristics of bacteria.

We explored the culturable bacterial community in the digestive tract of *S. litura* using a culture-dependent technique based on 16S rRNA gene sequencing and screening of these four isolates. Bacterial isolation was performed on living larvae separately. The larva was homogenized in nutrient extract using a glass pounder, and the homogenate is filtered 2 times to remove larvae debris than input into sterile tubes. The larvae extract a number of 50 µL were placed on nutrient agar and incubated at 37°C in a humidified atmosphere containing at 5% CO_2_ moisture and allowed to increase the number of bacteria for 3 days. Isolates were distinguished based on colony color and morphology. After that, the pure cultures of bacterial colonies were added into 20% glycerol and prepared at the Laboratory of Microbiology of the Department of Microbiology, Faculty of Veterinary Medicine, Airlangga University.

Identification of bacterial isolates was identified by various tests, such as the utilization of organic compounds, spore formation, Gram staining, NaCl tolerance, optimum temperature, optimum pH, and catalase [4].

The isolate identification of four bacteria was confirmed using 16S rRNA gene sequencing. The standard protocol was used for confirm of total genomic DNA extraction. The isolated DNAs of each bacteria, i.e., *Bacillus* sp.*, Cellulomonas* sp.*, Pseudomonas* sp., and *Cytophaga* sp. were stored at −20°C until use. Furthermore, the polymerase chain reaction (PCR) amplification of the 16S rRNA genes was performed using the universal primers UNI16S-L (5’-ATTCTAGAGTTTGATCATGGCTCA-3’) as the forward primer and UNI16S-R (5’-ATGGTACCGTGTGA CGGGCGGTGTGTA-3’) as the reverse primer and then Amplification process in a thermocycler (Eppendorf, Mastercycler Gradient, Hamburg, Germany) for 36 reaction cycles. Reactions were routinely performed in 50 µL including 1.5 µL of 10 mM dNTP mix, 1.5 µL of 10 pmol each of the opposing amplification primers, 1 µL of 5 U/µL Taq DNA polymerase (Fermentas), 3 µL of MgCl2, 5 µL of Taq DNA polymerase reaction buffer, 1 µL of genomic DNA, and 35.5 µL of dH2 O. PCR conditions were 5 min at 95°C for the initial denaturation of template DNA, 36 amplification cycles (1 min at 94°C, 1 min at 56°C, and 2 min at 72°C), and 10 min at 72°C for the final extension. PCR products were separated on 1.0% agarose gels, stained with ethidium bromide, and viewed under ultraviolet light. After checking the PCR products, they were sent to Macrogen (the Netherlands) for sequencing. The obtained sequences were used to perform BLAST searches using the NCBI GenBank database. In addition, sequences were used for phylogenetic analysis for further characterization [9].

#### Second stage

The second stage, the process of soybean fermentation from Tempe industry waste (Usaha Tempe Rakyat, Surabaya, Indonesia), with the addition of Epidopt (Sugar Factory Candi, Sidoarjo, Indonesia), urea (Petrokimia Gresik, Gresik, Indonesia), and various bacterial isolates obtained from Stage 1 studies compared with control (without addition of bacterial isolate). Fermentation is one of the major processes used in the production of food from soybeans. This fermentation changes the physicochemical and organoleptic properties of soy products such as color, flavor, and active components [10].

The second stage used complete randomized design with 5 treatments and 4 replicates [11]. The treatment was: T0: Soybean husk + 1% molasses + 1% urea + without bacterial isolate; T1: Soybean husk + 1% molasses + 1% urea + 5% bacillus sp. bacterial isolate; T2: Soybean husk + 1% molasses + 1% urea + 5% bacteria *Cellulomonas* sp. isolate; T3: Soybean husk + 1% molasses + 1% urea + 5% pseudomonas sp. bacterial isolate; and T4: Soybean husk+ 1% molasses + 1% urea + 5% *Cytophaga* sp. bacterial isolate.

A total of 20 samples of soybean husk, each weighing 200 g, were randomly divided into 5 treatments with 4 replicates, 1% urea + epidopt and 5% of cellulolytic bacteria (108/cc) dissolved in a diluent solution of sterile water as much as 30% of the sample weight. Subsequently, the solution was sprayed on the husk of the soybeans and inserted into a plastic bag (clear, hollow in some places, and tied at the top), and fermented for 7 days. After the fermentation process ended, the organoleptic examination was done, including color, odor, texture, and pH measurement. Then, the fermented husk was aerated. Furthermore, to determine the content of DM, crude fiber, and CP, the proximate analysis was performed according to the method recommended by Sruamsiri and Silman [1]. The best results of this second stage were T2: Soybean husk + *Cellulomonas* sp. suspension (1% Molasses + 1% urea + 5% isolate *Cellulomonas* sp. as fermenter).

#### Third stage

The third stage of this study was the application of a complete feed formulation by adding fermentation of the best result of the second stage: Various percentage of soybean husk + *Cellulomonas* sp. suspension, compared with control (without *Cellulomonas* sp. suspension). Furthermore, prepared complete feed formulation was given as feed on the *Anas javanica*. The complete feed formulation is shown in Table-1.

**Table-1:** Complete feed formulation was given to *Anas javanica* using soybean husk waste fermented with *Cellulomonas* sp. bacteria suspension.

Materials (%)	T0 (Control) complete feed without soybean husk and *Cellulomonas* sp. bacteria suspension	T1 (Treatment 1) complete feed+15% soybean husk without *Cellulomonas* sp. bacteria suspension	T2 (Treatment 2) complete feed+15% soybean husk+0.05% *Cellulomonas* sp. bacteria suspension	T3 (Treatment 3) complete feed+30% soybean husk without *Cellulomonas* sp. bacteria suspension	T4 (Treatment 4) complete feed+30% soybean husk+0.05% *Cellulomonas* sp. bacteria suspension
Yellow corn	61.00	46.00	46.00	31.00	31.00
Fish meal	13.80	13.75	13.80	13.80	13.75
Soy meal	5.60	5.60	5.60	5.60	5.60
Rice bran	14.70	14.70	14.70	14.70	14.70
Soybean	4.30	4.30	4.30	4.30	4.30
Coconut oil	0.30	0.30	0.30	0.30	0.30
Premix	0.30	0.30	0.30	0.30	0.30
Soybean husk	-	15	15	30	30
*Cellulomonas* sp. bacteria suspension	-	-	0.05	-	0.05
Total	100	100	100	100	100

The third stage of this study was giving complete feed formulation to *Anas javanica* in improving the quality of *Anas javanica* egg. This study used 100 laying *Anas javanica*, aged about 20 weeks, divided into 5 treatments in the form of 5 types of formula feed which were T0: Complete feed without soybean husk and *Cellulomonas* sp. bacteria suspension; T1: Complete feed + 15% soybean husk without *Cellulomonas* sp. bacteria suspension; T2: Complete feed + 15% soybean husk + 0.05% *Cellulomonas* sp. bacteria suspension; T3: Complete feed + 30% soybean husk without *Cellulomonas* sp. bacteria suspension; and T4: Complete feed + 30% soybean husk + 0.05% *Cellulomonas* sp. bacteria suspension (Table-1). The experimental design was complete randomized design (5×5 replicates). Parameters to improve the quality of *Anas javanica* eggs included egg cholesterol levels, egg weight, egg yolk, and eggshell thickness. Egg cholesterol (mg/100 g) levels were measured on day 7 before the end of the study. Cholesterol levels were tested using the Liebermann–Burchard’s method [12,13]. Egg weight is measured by weighing using digital scales. Evaluation of egg yolk color estimated by the usual method applying La Roche scale (DSM Yolk Color Fan) with spectrophotometric determination of β-carotene by AOAC method, and by new rapid analyzer iCheckTM Egg photometer (BioAnalyt). The yolk color varied between the values of 4 and 13 of La Roche scale. The carotenoid content expressed as β-carotene measured by AOAC method varied between 11 and 87 mg/kg. The carotenoid content expressed as β-carotene measured with the analyzer Check TM Egg photometer was lower and varied between 7.5 and 68.5 mg/kg [14].

The measurements of eggshell thickness were done using ultrasonography technology. The measurements beginning from the large end to small end of the egg and repeated at each on 3 meridians in parallel. The measurements were taken with an electronic micrometer measurement predominantly at the wider end of eggs [15].

### Statistical analysis

Cholesterol, egg weight, and eggshell thickness were statistically analyzed using SPSS 13 for Windows XP with the confidence level of 99% (α=0.01) and the level of significance 0.05 (p=0.05). Hypothesis tests were as follows: Normality test of the data with Kolmogorov–Smirnov test, homogeneity of variance test, analysis of variance, and *post hoc* test using Tukey test with very significant difference 5% [16].

## Results

### Isolation and identification of cellulolytic bacteria of *S. litura*

The results of isolation and identification of the digestive tract of *S. litura*, which was the first stage of this study, it is found of 4 isolates of cellulolytic bacteria, they are *Bacillus* sp., *Cellulomonas* sp., *Pseudomonas* sp., and *Cytophaga* sp. Furthermore, the four isolates were, respectively, used as fermenters on the soybean husk from Tempe industry wastes derived from Usaha Tempe Rakyat Surabaya, Indonesia, at the next stage of the study.

### Improving the quality of soybean husk waste

Improving the quality of soybean husk waste, which is the second stage of this research, is done through fermentation process with the addition of epidopt (Sugar Factory of Candi, Sidoarjo, Indonesia), urea (Petrokimia Gresik, Gresik, Indonesia), and various bacterial isolates which was obtained from first stage of the study (*Bacillus* sp., *Cellulomonas* sp., *Pseudomonas* sp., and *Cytophaga* sp.**) and compared with control (without addition of bacterial isolates). The results of this second stage study can be seen in Table-2.

**Table-2:** The content of dry material (%), crude fiber (%), and CP (%) of fermented soybean husk using various cellulolytic bacteria isolates from *S. litura*.

Treatment	Dry material (%)±SE	Crude fiber (%)±SE	CP (%)±SE
T0 (soybean husk+1% molasses+1% urea+without bacteria isolate)	78.15^a^±0.05	48.60^a^±0.14	15.63^a^±0.26
T1 (soybean husk+1% molasses+1% urea+5% *Bacillus* sp. bacteria isolate)	78.79^a^±0.82	48.73^a^±0.53	15.85^a^±0.73
T2 (Soybean husk+1% molasses+1% urea+5% *Cellulomonas* sp. bacteria isolate)	78.18^a^±0.20	43.81^b^±0.78	15.90^a^±0.55
T3 (soybean husk+1% molasses+1% urea+5% *Pseudomonas* sp. bacteria isolate)	78.67^a^±0.16	48.07^a^±0.50	17.10^b^±0.90
T4 (soybean husk+1% molasses+1% urea+5% *Cytophaga* sp. bacteria isolate)	78.40^a^±0.19	48.58^a^±1.38	17.57^b^±0.68

a,b,c Values in the same column with different superscripts indicate significant difference *p<*0.05 (n=4), CP=Crude protein, *S. litura=Spodoptera litura*, SE=Standard error

### The eggs quality of *Anas javanica*

The egg’s quality of *Anas javanica* after feeding with a wide variety of complete feeds (both with the addition of soybean husk and the suspension of cellulolytic bacteria) compared with no addition was observed through cholesterol levels from egg yolks, egg weight, eggshell thickness, and egg yolk color.

### The cholesterol eggs level of duck

The cholesterol eggs level (mg/100 g) based on Liebermann–Burchard’s method [12,13] was measured on day 7 before the end of the study. The mean and standard deviation of cholesterol eggs levels of duck which is given with various feeding complete either by addition of soybean husk fermented using suspension of *Cellulomonas* sp. bacteria from *S. litura* was compared with not addition. The cholesterol eggs level can be seen in Table-3.

**Table-3:** Mean and standard deviation of egg yolk cholesterol levels of *Anas javanica*.

Treatments	Mean±SE
T0 (complete feed without soybean husk and *Cellulomonas* sp. bacteria suspension)	18.74^a^±2.19
T1 (complete feed+soybean husk 15% without *Cellulomonas* sp. bacteria suspension)	15.61^b^±2.12
T2 (complete feed+soybean husk 15% + 0.05% *Cellulomonas* sp. bacteria suspension)	16.53^b^±2.52
T3 (complete feed+soybean husk 30% without *Cellulomonas* sp. bacteria suspension)	13.35^c^±1.92
T4 (complete feed+soybean husk 30% + 0.05% *Cellulomonas* sp. bacteria suspension.)	12.69^c^±2.23

a,b,c Values in the same column with different superscripts indicate significant difference *p<*0.05 (n=5), SE=Standard error

### Egg weight, eggshell thickness, and egg yolk color

Mean, and standard deviation of duck egg quality, including egg weight, eggshell thickness, and egg yolk color which is given with various feeding complete either by addition of soybean husk waste fermentation using suspension of *Cellulomonas* sp. cellulose from *S. litura* was compared with not addition. Egg weight, eggshell thickness, and egg yolk color can be seen in Table-4.

**Table-4:** Mean and standard deviation of egg weight, egg yolk color, and egg shell thickness of duck.

Variable	Treatment				

	T0 (complete feed without soybean husk and *Cellulomonas* sp. bacteria suspension)	T1 (complete feed+15% soybean husk without *Cellulomonas* sp. bacteria suspension)	T2 (complete feed+15% soybean husk+0.05% *Cellulomonas* sp. bacteria suspension)	T3 (complete feed+30% soybean husk without *Cellulomonas* sp. bacteria suspension)	T4 (complete feed+soybean husk 30% + 0.05% *Cellulomonas* sp. bacteria suspension)
Egg weight (g)±SE	47.60^a^±4.07	50.96^a^±3.38	52.26^a^±2.48	42.17^a^±20.12	47.94^a^±9.37
Egg yolk color±SE	10.20^b^±1.79	8.20a^b^±1.92	9.40^b^±2.30	8.00^ab^±1.41	6.40^a^±2.30
Eggshell thickness (mm)±SE	0.55^a^±0.08	0.52^a^±0.08	0.53^a^±0.03	0.53^a^±0.07	0.53^a^±0.08

a,b,c Values in the same line with different superscripts indicate significant difference *p<*0.05 (n=5), SE=Standard error

## Discussion

Based on the results of variance analysis, it was found that the content of crude fiber and CP of soybean husk fermentation using 4 bacterium: *Bacillus* sp., *Cellulomonas* sp., *Pseudomonas* sp., and *Cytophaga* sp. have shown significantly different results (p<0.05), while the content of dry material was not showed a significant difference (p>0.05). Based on Duncan’s distance test for crude fiber content, the best result, the highest decrease of the crude fiber, was in T2 treatment, which was treated with a suspension of *Cellulomonas* sp.

According to Holt [17], the bacteria *Cellulomonas* sp. is Gram-positive, rod-shaped, and non-motile. The characteristic of this bacterium is as follows: Respiratory metabolism using oxygen as electron acceptor, catalase positive, lives at optimum temperature 300°C, and neutral pH, with growth rate 0.15-0.23/h. These bacteria have been known to digest cellulose, xylene, and starch. According to Gupta *et al*. [18], *Cellulomonas* sp. possesses extracellular enzymes that play a greater role in the breakdown of amorphous cellulose.

Observations on cholesterol levels were showed that feeding complete in T0, which produces the highest cholesterol levels and significantly different (p<0.05%) than T1, T2, and T3. The feeding complete in T4 has yielded the lowest cholesterol level compared with T3 treatment but significantly different (p<0.05%) with T1 and T2 treatment, whereas between T1 and T2 treatment did not significantly different (p> 0.05%). This result provides an opportunity to the utilization of complete feed with the addition of fermented soybean husk using *Cellulomonas* sp. bacteria suspension from which gives the best result as the lowest cholesterol level.

Several other studies, such as the provision of katuk leaf flour which also contains high crude fiber as well as soybeans husk, showed that katuk leaf flour at level ≥5% was also able to decrease cholesterol levels of eggs Mojosari duck without decreasing percentage of egg yolk weight [19]. However, since the use of katuk leaf flour must compete with food consumed by humans, the utilization of the soybean husk waste can be an alternative to consider. Furthermore, in many other studies on the use of various foliage powders with a high content of crude fiber, egg cholesterol levels of duck cannot be reduced. A study conducted by Palupi *et al*. [20], eggs cholesterol level of duck with an additional meal of beluntas leaves up to 2%; the level had no effect on egg cholesterol level of duck, where cholesterol levels at the treatment were still at 27.79 mg/g egg yolks.

Table-4 shows that egg yolk color parameters on T0, T1, T2, and T3 treatments result in a significantly different color of egg yolk (p<0.05), whereas T4 yields a lower yolk color than the other four treatments. This shows that the provision of soybean husk fermentation from Tempe industry waste as much as 30% as a substitute for corn can affect the color of egg yolks. Subhan [21] reported that the score of egg yolk color of the *Anas javanicus* from Tegal region, Indonesia was < 7.5, while Beardsworth and Hernandez [22] stated that the good egg yolk color was in the range of 8-12. The good egg yolk color in the range of 8-12 was obtained with the addition of corn to the feed. Corn is one of the agricultural commodities very important for livestock. Corn is a high-energy feed ingredient, with a protein content of about 8.6-9.0%, but corn protein cannot ferment or degraded by rumen microorganisms [23].

The parameter observation of egg weight and eggshell thickness was not showed a significant difference between treatments (p>0.05). This shows that the utilization of soybean husk waste fermented with *Cellulomonas* sp. bacteria up to 30% dose does not affect egg weight or eggshell thickness. Silversides and Villeneuve [24] reported that with increasing age, the egg size would increase as a result of increased yolk weight.

In poultry, including ducks, the process of egg formation known as folliculogenesis, in addition to affecting the development of the oocyte (egg cell), also affects the weight of the egg yolk. The number of follicles during one cycle is influenced by factors such as animal species, reproductive phase, circumstances, age, mother, genetic [25], and feed [26-28].

## Conclusion

The fermentation of soybean husk from Tempe industry waste through the utilization of cellulolytic bacteria of *S. litura* added to complete feed can be done as an effort to improve the quality of *Anas javanic*a eggs in the form decrease of egg yolks cholesterol level without decreasing egg weight and eggshell thickness, although the decrease in yolk color is unavoidable statistically does not show significant differences (p>0.05).

## Authors’ Contributions

All the authors conceptualized the manuscript. SH and ES drafted the manuscript. SH: Research project leader and coordinating research, collected and processed samples. Carried out the data collection and gathering assay samples. DSN has done the statistical analysis part and critically reviewed the manuscript. ES: Assisted in manuscript preparation and corresponding author. All the authors have read and approved the final version of the manuscript.
